# The prevalence and origin of exoprotease-producing cells in the *Bacillus subtilis* biofilm

**DOI:** 10.1099/mic.0.072389-0

**Published:** 2014-01

**Authors:** Victoria L. Marlow, Francesca R. Cianfanelli, Michael Porter, Lynne S. Cairns, J. Kim Dale, Nicola R. Stanley-Wall

**Affiliations:** 1 Division of Molecular Microbiology, College of Life Sciences, University of Dundee, Dundee DD1 5EH, UK; 2 Centre for Gene Regulation and Expression, College of Life Sciences, University of Dundee, Dundee DD1 5EH, UK; 3 Division of Cell and Developmental Biology, College of Life Sciences, University of Dundee, Dundee DD1 5EH, UK

## Abstract

Biofilm formation by the Gram-positive bacterium *Bacillus subtilis* is tightly controlled at the level of transcription. The biofilm contains specialized cell types that arise from controlled differentiation of the resident isogenic bacteria. DegU is a response regulator that controls several social behaviours exhibited by *B. subtilis* including swarming motility, biofilm formation and extracellular protease (exoprotease) production. Here, for the first time, we examine the prevalence and origin of exoprotease-producing cells within the biofilm. This was accomplished using single-cell analysis techniques including flow cytometry and fluorescence microscopy. We established that the number of exoprotease-producing cells increases as the biofilm matures. This is reflected by both an increase at the level of transcription and an increase in exoprotease activity over time. We go on to demonstrate that exoprotease-producing cells arise from more than one cell type, namely matrix-producing and non-matrix-producing cells. *In toto* these findings allow us to add exoprotease-producing cells to the list of specialized cell types that are derived during *B. subtilis* biofilm formation and furthermore the data highlight the plasticity in the origin of differentiated cells.

## Introduction

The formation of sessile communities of microbial cells called biofilms is a process common to many bacterial strains (Costerton *et al.*, 1995). The resultant biofilm communities can have both beneficial and detrimental impacts on human society and are linked with processes as diverse as bioremediation and chronic infections (Costerton *et al.*, 1987). Biofilm formation is underpinned by the production of an extracellular matrix that is commonly composed of DNA, proteins and exopolysaccharides (Flemming & Wingender, 2010). The extracellular matrix generates and stabilizes the 3D structure and provides protection to the resident bacteria (Branda *et al.*, 2005).

*Bacillus subtilis* is a Gram-positive soil-dwelling bacterium used as a model for biofilm formation (Vlamakis *et al.*, 2013). The biofilm matrix is composed of TasA amyloid-like fibres (Branda *et al.*, 2006; Romero *et al.*, 2010), a secreted exopolysaccharide (Branda *et al.*, 2001; Chai *et al.*, 2012) and a bacterial hydrophobin called BslA that forms a hydrophobic coat over the biofilm (Hobley *et al.*, 2013; Kobayashi & Iwano, 2012; Ostrowski *et al.*, 2011). Synthesis of the components within the biofilm extracellular matrix is tightly regulated at the level of transcription (Vlamakis *et al.*, 2013). In *B. subtilis*, one key regulator that is required for biofilm formation, due to its role in controlling the biosynthesis of the BslA coat protein, is DegU (Kobayashi, 2007; Ostrowski *et al.*, 2011). DegU is the response regulator of the DegS–DegU two-component regulatory system (Dahl *et al.*, 1991). Phosphorylated DegU (hereafter DegU-P)-regulated processes are upregulated in response to several environmental signals (for a review see Murray *et al.*, 2009a) but of particular relevance to biofilm formation the system is activated when rotation of the flagella is impeded (Cairns *et al.*, 2013).

*B. subtilis* biofilm formation is hallmarked by the differentiation of genetically identical cells within the population into specialist subtypes (Branda *et al.*, 2001; Vlamakis *et al.*, 2008). To date, cells specialized towards motility, biofilm matrix production and sporulation have been identified (Vlamakis *et al.*, 2008). However, the occurrence and origin of cells that produce extracellular proteases (hereafter exoproteases) within the biofilm have not been examined. The production of exoproteases by *B. subtilis* occurs heterogeneously in planktonic culture (Veening *et al.*, 2008) and transcription is dependent on DegU-P (Dahl *et al.*, 1992; Tsukahara & Ogura, 2008). Moreover, exoprotease activity has been shown to be required for pellicle formation in laboratory isolates of *B. subtilis* (Connelly *et al.*, 2004). Therefore, given this knowledge, and the known role for DegU-P in controlling biofilm formation (Kobayashi, 2007; Stanley & Lazazzera, 2005; Verhamme *et al.*, 2007), here we define the impact of changing DegU-P levels on the proportion of cells in the biofilm population that transcribe the genes required for exoprotease synthesis. We identify that as biofilm formation progresses, exoprotease production increases at the level of both transcription and activity. Using live cell microscopy analysis of microcolony formation, we assess the origin of the exoprotease-producing cells and identify that they arise from both matrix-producing cells and non-matrix-producing cells. These findings shed light on the diversity of specialized cell types contained within the biofilm and highlight plasticity in their origin.

## Methods

### 

#### Growth conditions.

The *Escherichia coli* and *B. subtilis* strains used and constructed in this study are detailed in Table 1. Both *E. coli* and *B. subtilis* strains were routinely grown in Luria–Bertani (LB) medium (per litre: 10 g NaCl, 5 g yeast extract and 10 g tryptone). Biofilm pellicles were grown in 10 ml MSgg medium (5 mM potassium phosphate and 100 mM MOPS at pH 7.0 supplemented with 2 mM MgCl_2_, 700 µM CaCl_2_, 50 µM MnCl_2_, 50 µM FeCl_3_, 1 µM ZnCl_2_, 2 µM thiamine, 0.5 % glycerol and 0.5 % glutamate) (Branda *et al.*, 2001) at 23 or 25 °C as defined, for up to 96 h. Complex colony biofilms were grown on MSgg solidified with 1.5 % Select Agar (Invitrogen) at 30 or 37 °C for the times indicated. Ectopic gene expression was induced with IPTG at the concentrations detailed. When appropriate, antibiotics were used at the following concentrations: ampicillin 100 µg ml^−1^, chloramphenicol 5 µg ml^−1^, kanamycin 25 µg ml^−1^, lincomycin 25 µg ml^−1^ with erythromycin 5 µg ml^−1^ and spectinomycin 100 µg ml^−1^.

**Table 1.** Strains and plasmids used in this study

Strain	Relevant genotype/description*	Reference, source or construction†
168	*trpC2*	BGSC
DS1993	NCIB3610 *degU* : : Tn*10* (MLS)	D. Kearns
NCIB3610	Wild-type (prototroph)	BGSC
NRS1314	NCIB3610 *degU* : : pBL204 (*cml*)	Verhamme *et al.* (2007)
NRS1325	NCIB3610 *amyE* : : P*hy-spank-degU hy 32* (*spc*) *degU* : : pBL204 *(cml)*	Verhamme *et al.* (2007)
NRS2313	168 P*bpr–gfp* (*cml*)	Veening *et al.* (2008)
NRS2315	NCIB3610 P*bpr*–*gfp* (*cml*)	NRS2313→NCIB3610
NRS2769	NCIB3610 *degU* : : Tn*10* (MLS) P*bpr*–*gfp* (*cml*)	DS1993→NRS2313
NRS2771	NCIB3610 *amyE *: : P*hy-spank-degU hy 32* (*spc*) *degU* : : Tn*10* (MLS) (*cml*) P*bpr*–*gfp* (*cml*)	NRS1311→NRS2769
NRS3372	168 *lacA* : : P*tapA*–*mCherry* (*erm*)	pNW702→168
NRS3373	NCIB3610 *lacA* : : P*tapA*–*mCherry* (*erm*)	NRS3372→NCIB3610
NRS3378	NCIB3610 P*bpr*–*gfp* (*cml*) *lacA* : : P*tapA*–*mCherry* (*erm*)	NRS2313→NRS3373
NRS3921	NCIB3610 P*bpr*–*gfp* (*cml*) *lacA* : : P*tapA*–*mKate2* (*erm*)	NRS3925→NRS2315
NRS3922	NCIB3610 *lacA* : : P*tapA*–*mKate2* (*erm*)	NRS3925→NCIB3610
NRS3925	168 *lacA* : : P*tapA*–*mKate2* (*erm*)	pNW726→NCIB3610

* Drug resistance cassettes are indicated as follows: *cml*, chloramphenicol resistance; *kan*, kanamycin resistance; *tet*, tetracycline resistance; MLS, lincomycin and erythromycin resistance; *spc*, spectinomycin resistance.

† The direction of strain construction is indicated with DNA or phage (SPP1) (→) recipient strain. BGSC, *Bacillus* Genetic Stock Center.

#### Strain construction.

*E. coli* strain MC1061 [F′*lacI*q *lacZ*M15 Tn*10* (*tet*)] was used for the construction and maintenance of plasmids. *B. subtilis* 168 derivatives were generated by transformation of competent cells with plasmids using standard protocols (Harwood & Cutting, 1990). SPP1 phage transductions, for introduction of DNA into *B. subtilis* strain NCIB3610 (hereafter 3610), were conducted as described previously (Verhamme *et al.*, 2007).

#### Plasmid construction

##### pNW700.

*mCherry* (711 bp) was amplified from pRSET-mCherry (kindly provided by Roger Y. Tsien, University of California, San Diego) using primers NSW1000 (5′-GGCCAAGCTTAAGGAGGTGATCATTAAAAATGGTGAGCAAGGGCGAGGAG-3′) and NSW1001 (5′-CGTAGGATCCTTACTTGTACAGCTCGTCCAT-3′). The resulting PCR product was digested with *Bam*HI and *Hin*dIII and inserted into pNW600 (Murray *et al.*, 2009b), digested the same way to replace the *gfp* coding region with the *mCherry* coding region yielding a P*tapA*–*mCherry* fusion in a vector that allows for integration at the non-essential *amyE* locus.

##### pNW702.

pNW700 was digested with *Eco*RI and *Bam*HI to release the P*tapA*–*mCherry* coding region, which was ligated into the *lacA* integration vector pDR183 which was digested the same way. This would enable integration at the non-essential *lacA* locus.

##### pNW725.

*mKate2* (746 bp) was amplified by PCR using the pTMN387 (kind gift of Professor Richard Losick, Harvard University) as the template and primers mKate-for (NRS1026) (5′**-**GTACAAGCTTAAGGAGGAACTACTATGGATTCAATAGAAAAGGTAAG-3′) and mKate-rev (NRS1027) (5-GTACGGATCCTTATCTGTGCCCCAGTTTGCT-3) (Chen *et al.*, 2013). The PCR product was digested with *Hin*dIII and *Bam*HI and ligated into plasmid pNW600 (Murray *et al.*, 2009b), which was cut the same way to yield the P*tapA*–*mKate2* reporter fusion in a vector that allows for integration at the non-essential *amyE* locus.

##### pNW726.

The P*tapA*–*mKate2* coding region was released from pNW725 by *Eco*RI and *Bam*HI digestion. The fragment was ligated into the *lacA* integration vector pDR183, which was digested the same way. This would enable integration at the non-essential *lacA* locus.

#### Biofilm formation assays.

Analysis of biofilm formation was performed as previously described (Branda *et al.*, 2001; Verhamme *et al.*, 2007).

#### Secreted protease activity assay.

Each 10 ml pellicle sample was collected by centrifugation (17 000 ***g*** for 10 min), after which the supernatant was removed and stored at −20 °C until use. The remaining cell pellet was used to determine the wet pellet weight. From 48 h onwards the cell pellet was resuspended in 10 ml double-distilled water (ddH_2_O) and subjected to gentle sonication (such that the cells did not lyse (Ostrowski *et al.*, 2011)), and the cell pellet was collected by centrifugation for 20 min, at 9000 ***g*** at 4 °C, prior to wet pellet weight analysis. To determine extracellular protease activity, the azocasein assay (Braun & Schmitz, 1980) was performed. A 150 µl aliquot of thawed supernatant was mixed with 500 µl of 2 % (w/v) azocasein (Sigma), along with 100 µl Tris-HCl (pH 8.0) and 650 µl ddH_2_O. A blank sample was prepared containing ddH_2_O in place of the supernatant and a medium-only control sample containing LB in place of the supernatant was also prepared. The samples were incubated for 1 h at 30 °C, after which 375 µl of 14 % (v/v) perchloric acid was added to stop each reaction. The samples were centrifuged (17 000 ***g*** for 5 min) and 750 µl of the supernatant was mixed directly in a cuvette with 75 µl of 10 M NaOH and the absorbance at 436 nm was measured using a spectrophotometer. The background activity of the medium-only control was subtracted and activity was calculated as Δ*A*_436_/h/ml/mg protein (Cairns *et al.*, 2013).

#### Flow cytometry.

The fluorescence of strains harbouring *gfp* promoter fusions was measured in single cells extracted from biofilm-forming conditions after incubation at either 30 or 37 °C as described previously (Murray *et al.*, 2009b; Vlamakis *et al.*, 2008).

#### Time-lapse microscopy.

Single colonies of *B. subtilis* were inoculated into 5 ml of MSgg medium and grown overnight at 30 °C and 220 r.p.m. The next morning cells were diluted 25-fold into 3 ml of 15 % MSgg medium. After approximately 4 h of incubation at 30 °C and 220 r.p.m., or when the cells had reached mid-exponential phase of growth, the sample was diluted to an OD_600_ of 0.007 in fresh 15 % MSgg medium. This enabled the visualization of single cells with the appropriate spacing for the start of the time-lapse acquisition. Then, 2 µl of this cell suspension was inoculated onto a thin matrix of 15 % MSgg supplemented with 1.5 % agarose (Invitrogen ultrapure agarose) on a microscope slide. Each slide was prepared as follows. A 125 µl Gene Frame (AB-0578; ABgene House) was attached to a standard microscope slide (VWR superpremium). The Gene Frame was next filled with molten 15 % MSgg supplemented with 1.5 % agarose (hereafter 15 % MSgg-agarose) with the addition of IPTG at the defined concentrations and covered firmly with a standard microscope slide to flatten the agarose surface. When the 15 % MSgg-agarose had sufficiently cooled and solidified the upper slide was carefully removed and the 15 % MSgg-agarose was carefully removed with a surgical scalpel blade (Swann Morton number 11) leaving behind either one or two strips of MSgg-agarose (~1.5 mm wide) in the centre of the Gene Frame. For experiments where two or more strips were required the strips were spaced at least 4 mm apart. de Jong *et al.* (2011) established that these conditions provide air cavities that are essential for efficient growth of *B. subtilis*. After inoculation the cell suspension was allowed to dry after which the Gene Frame was sealed with a coverslip (22×22 mm; VWR) 1.5 mm thick. The microscope slides were incubated at 30 °C in a temperature-controlled environmental chamber (Weather Station; Applied Precision). Prior to the start of acquisition the cells were allowed to equilibrate on the agarose pads for 3 h. Time-lapse imaging of microcolony development and P*tapA*–*mKate* and P*bpr*–*gfp* expression was performed using a DeltaVision Core wide field microscope (Applied Precision) mounted on an Olympus IX71 inverted stand with an Olympus ×60, 1.4 NA lens and CoolSNAPHQ camera (Photometrics) with differential interference contrast (DIC) and fluorescence optics. For each experiment 12 independent fields were manually identified and their *XYZ*-positions were stored in the microscope control software (SoftWorx; Applied Precision). Images (512×512 pixels with 2×2 binning and 12 *Z* sections spaced at 1 µm) were acquired every 15 min for up to 12 h. GFP was imaged using a 100 W mercury lamp and an FITC filter set (excitation 490/20; emission 528/38) with an exposure time of 200 ms. mKate2 was imaged using a 100 W mercury lamp and a TRITC filter set (excitation 555/28; emission 617/73) with an exposure time of 300 ms. DIC images were acquired with an LED transmitted light source (Applied Precision) at 32 % intensity and exposure times between 25 and 50 ms. Post-acquisition images were rendered and analysed using omero software (http://openmicroscopy.org) (Allan *et al.*, 2012). The threshold used to define activation of the transcriptional reporter was set as a fluorescence intensity value greater than two standard deviations above the mean background fluorescence.

#### Microscopy of cells harvested from complex colonies

##### DIC microscopy and fluorescence microscopy.

Colony biofilms were grown as before (Branda *et al.*, 2001; Verhamme *et al.*, 2007) and harvested as previously described for flow cytometry (Murray *et al.*, 2009b; Vlamakis *et al.*, 2008) with the exception that the cells were not fixed. After washing in 1× PBS the cells were diluted 10-fold into GTE buffer (50 mM glucose, 10 mM EDTA at pH 8, 20 mM Tris/HCl at pH 8), 2 µl of the cell suspension was spotted onto a 1.5 % agarose pad and images were acquired using a DeltaVision Core wide field microscope (Applied Precision) mounted on an Olympus IX71 inverted stand with an Olympus ×100, 1.4 NA lens and Cascade2 512 EMCCD camera (Photometrics). Images (512×512 pixels with 13 *Z* sections spaced at 0.2 µm) were acquired with DIC and fluorescence optics. GFP and mCherry were detected using a 100 W mercury lamp and an FITC filter set (excitation 490/20; emission 528/38) and a TRITC filter set (excitation 555/28; emission 617/73), respectively. DIC images were acquired with an LED transmitted light source (Applied Precision) at 32 % intensity and exposure times between 25 and 50 ms. Post-acquisition images were rendered and analysed using omero software (http://openmicroscopy.org) (Allan *et al.*, 2012). All figures were assembled in Canvas 12 (ACD Systems).

##### Phase-contrast and fluorescence microscopy.

Colony biofilms were grown and harvested as for DIC and fluorescence microscopy and prepared for imaging as described above. Microscopy was performed using a ×100 Plan-NEOFLUAR 1.30 oil immersion lens on an Axio Imager M1 microscope mounted with an Axiocam MRm camera (Zeiss). GFP fluorescence was visualized using an FITC filter set (excitation 490/40; emission 525/50) and images were rendered and analysed using the AxioVision Rel. 4.8 (Zeiss) software. All figures were assembled in Canvas 12 (ACD Systems).

#### Biofilm sectioning and confocal microscopy.

Complex colonies formed by strain NRS3921 were grown on MSgg solidified with 1.5 % agar as described above. A quarter section of the colony (after 48 h growth) was excised with a no. 10 surgical scalpel and placed into O.C.T. compound (Agar Scientific) and frozen in iso-pentene chilled with liquid nitrogen. Cross-sections (9 µm) of the colony were cut using a Leica CM3050S cryomicrotome. The sections were transferred onto SuperFrost Ultra Plus adhesion microscope slides (VWR). Each section was fixed with 150 µl of 4 % para-formaldehyde in PBS for 10 min. The sections were then washed three times with Tris-buffered saline. A drop of mounting medium was applied onto the slide containing the colony sections (modified from Hobley *et al.*, 2013), onto which a 1.5 mm thick coverslip was placed. After removal of excess mounting medium the cover glass was sealed with nail varnish. The slides were stored at −20 °C prior to analysis. Samples were imaged using a Zeiss LSM710 confocal scanning laser microscope fitted with 488 and 594 nm lasers and a planApo ×25/0.8 NA oil objective. During each experiment the laser settings, scanning speed, photomultiplier gains and pinhole settings were kept constant for all acquired images. Images were captured using Zen2011 software and image analysis was conducted using the omero platform (www.openmicroscopy.org) (Allan *et al.*, 2012). All figures were assembled in Canvas 12 (ACD Systems).

## Results and Discussion

### An increase in transcription of the exoprotease-encoding *bpr* gene is observed at the single-cell level in the presence of high DegU-P

**Fig. 1.**
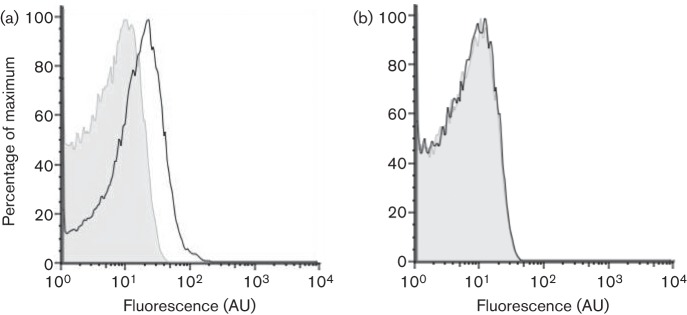
Transcription of the exoprotease-encoding *bpr* gene observed at the single cell level is dependent on DegU-P. Flow cytometry analysis of 3610 P*bpr*–*gfp* (NRS2315) (black line) (a) and 3610 P*bpr*–*gfp degU* (NRS2769) (black line) (b) using the parental 3610 strain as a negative control (grey-shaded area). Cells were grown under biofilm formation conditions for 17 h at 37 °C. A representative example is shown from three independent experiments.

The two major exoproteases synthesized by *B. subtilis* are subtilisin and bacillopeptidase, which are encoded by the *aprE* and *bpr* genes, respectively (Msadek, 1999). We followed exoprotease production using a P*bpr*–*gfp* reporter construct that was integrated at the native location on the chromosome (Veening *et al.*, 2008). The P*bpr*–*gfp* construct was introduced into NCIB3610 wild-type and *degU* mutant strains (Table 1) and transcription was monitored using flow cytometry and single-cell microscopy based on detection of GFP. Transcription from the *bpr* reporter fusion in the wild-type biofilm colony was assessed after 17 h of incubation. All the cells exhibited a low and homogeneous level of expression (Fig. 1a). Analysis confirmed that expression from the *bpr* promoter was DegU-dependent as the level of fluorescence decreased to the background basal level in the presence of a mutation in *degU* (Fig. 1b). These findings demonstrate that the transcriptional reporter behaved as expected during biofilm formation and in the NCIB3610 isolate of *B. subtilis* used here.

**Fig. 2.**
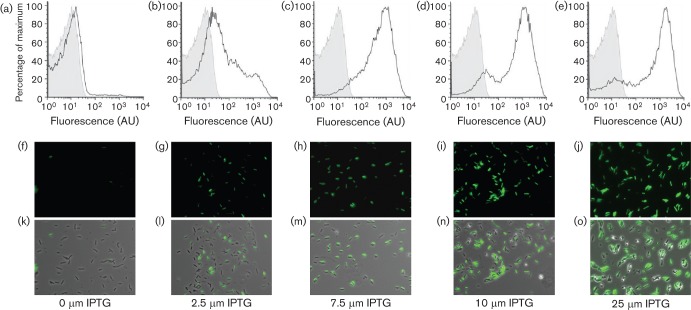
An increase in transcription of the exoprotease-encoding *bpr* gene is observed at the single-cell level in the presence of high DegU-P. (a–e) Flow cytometry analysis. The grey-shaded area represents the parental 3610 strain as a negative control and the black lines the experimental sample. (f–o) Microscopy of P*bpr*–*gfp degU*, *amyE* : : P*hy-spank-degU32 hy* cells (NRS2771). (f–j) Fluorescence was imaged in the FITC channel to detect GFP production. (k–o) The same cells analysed by phase-contrast fluorescence microscopy showing the overlay with the GFP expression with the cells. The cells were grown under biofilm formation conditions for 17 h at 37 °C in the presence of 0 µM IPTG (a, f, k), 2.5 µM IPTG (b, g, l), 7.5 µM IPTG (c, h, m), 10 µM IPTG (d, i, n) and 25 µM IPTG (e, j, o) prior to collection. In each case, one representative example is presented from three independent experiments.

We next investigated the impact of increasing levels of DegU-P on transcription from the *bpr* promoter element to establish if this would promote heterogeneity in *bpr* transcription. To control the level of DegU-P in the cell, the *degU32 hy* mutant allele of the *degU* coding region was introduced into the chromosome under control of the IPTG-inducible promoter (P*hy-spank* using pDR111) at the heterologous *amyE* locus (Verhamme *et al.*, 2007). This variant of DegU carries a histidine to leucine mutation in amino acid 12 and exhibits a lower level of dephosphorylation than wild-type DegU (Dahl *et al.*, 1991). The strain additionally contained a deletion of the native copy of *degU* and carried a P*bpr*–*gfp* reporter fusion at the native *bpr* locus on the chromosome (Table 1). Note that as transcription of *degU32 hy* is increased, biofilm formation itself is inhibited (Verhamme *et al.*, 2007). Both flow cytometry and fluorescence microscopy analysis demonstrated that consistent with DegU-P activating transcription from the *bpr* promoter region the number of GFP-positive cells increased with the addition of up to 25 µM IPTG to the growth medium (Fig. 2). The number of GFP-positive cells increased from 7 % in the absence of IPTG (Fig. 2a, f, k) to 84 % in the presence of 7.5 µM IPTG (Fig. 2e, j, o). The GFP-positive cells could be divided into low expression (10^1^–10^2^ AU) and high expression (10^2^–10^4^ AU) cells with the number of cells within the high expression category increasing alongside the level of DegU-P. Thus, it is evident that transcription from the *bpr* promoter can be highly heterogeneous in the biofilm population and that this is correlated with increases in the level of DegU-P.

### The level of exoprotease production increases during biofilm formation

**Fig. 3.**
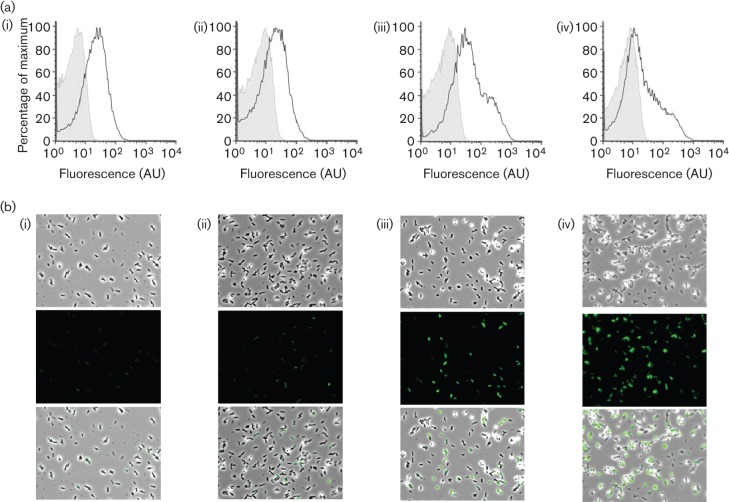
Transcription of *bpr* increases over time during biofilm formation. (a) Transcription of *bpr* from cells extracted from complex colonies was monitored over a 72 h period using a *bpr*–*gfp* transcriptional fusion strain (NRS2315). Colonies were grown at 30 °C and collected for flow cytometry analysis (grey-shaded area, non-fluorescent control 3610 strain; black line, *bpr*–*gfp*) after 17 h (i), 24 h (ii), 48 h (iii) and 72 h (iv). (b) The same cells were then analysed by phase-contrast fluorescence microscopy after 17 h (i), 24 h (ii), 48 h (iii) and 72 h (iv). Shown are the phase-contrast (top) and FITC (GFP) channel (middle) and an overlay of both channels (bottom). A representative example is presented from three independent experiments.

**Fig. 4.**
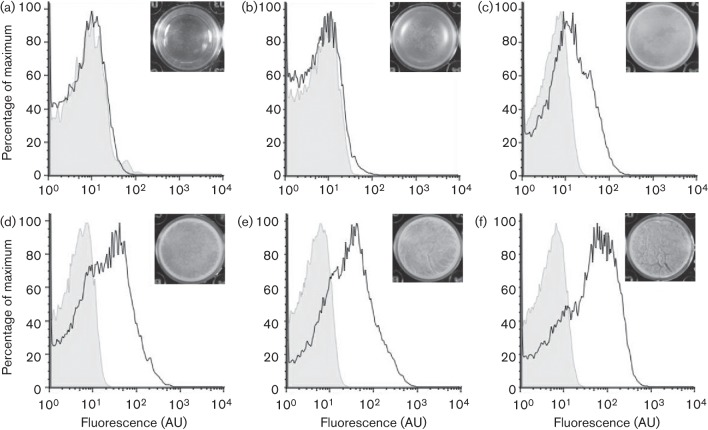
An increase in transcription of the exoprotease-encoding *bpr* gene occurs during pellicle formation. Transcription of *bpr* from cells extracted from pellicle biofilms was monitored over a 96 h period using a *bpr*–*gfp* transcriptional fusion strain (NRS2315). Transcription of *bpr* from cells extracted from pellicles grown at 23 °C was monitored over a 96 h period using a *bpr*–*gfp* transcriptional fusion. Flow cytometry data are shown from cells extracted from pellicles at 24 h (a), 36 h (b), 48 h (c), 60 h (d), 72 h (e) and 96 h (f). The non-fluorescent NCIB3610 control line is shown as the grey-shaded area and the experimental sample as a black line. A representative example of both the expression analysis and pellicle formation is presented from three independent experiments.

We next assessed if changes in the frequency of exoprotease transcription occurred over time during biofilm formation. Increases in exoprotease production would indicate an increase in the level of DegU-P during biofilm formation. To test this, transcription from the P*bpr*–*gfp* reporter construct in the wild-type strain was assessed at the single-cell level from samples isolated from complex colony and pellicle biofilms over a time-course of development, namely 17, 24, 48 and 72 h for colony biofilms (Fig. 3) and 24, 36, 48, 60, 72 and 96 h for pellicle biofilms (Fig. 4). Representative pellicle biofilm images at the point of collection are presented as inserts within Fig. 4. Flow cytometry and single-cell microscopy analysis of the disrupted biofilms revealed that transcription from the *bpr* promoter region increased during biofilm development for both biofilm types (Figs 3 and 4); moreover, transcription became highly heterogeneous as the biofilm matured [compare Fig. 3a(i) with 3a(iv) and Fig. 4c with 4d, e and f]. As reported above (Fig. 2), the GFP-positive cells in the biofilm could be subdivided into low (10^1^–10^2^ AU) and high (10^2^–10^3^ AU) expressing cells with the number of individual cells within the high expression category increasing over time. In fact, the peak in exoprotease transcription occurred at 72 h for the colony biofilm and 96 h for the pellicle biofilm. These findings demonstrate that transcription from the *bpr* promoter increases over time during biofilm formation.

**Fig. 5.**
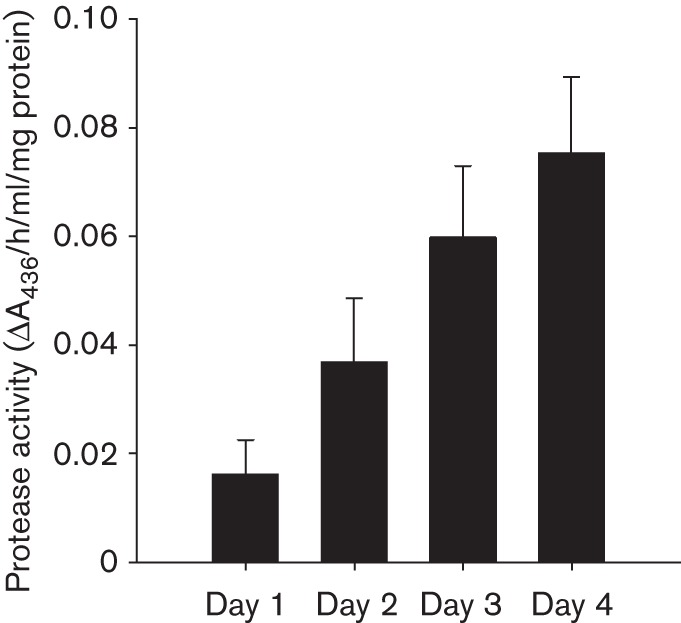
Protease secretion was analysed from supernatant collected from pellicles during biofilm formation. Protease secretion was assessed using an azocasein assay from pellicle supernatants. Pellicles were grown at 25 °C for up to 96 h and samples were collected at 24 h (day 1), 48 h (day 2), 72 h (day 3) and 96 h (day 4). Enzyme activity was normalized against wet pellet weight of the pellicle. Data are presented as the mean of four independent experiments and the error bars represent sem.

To correlate *bpr* transcription with exoprotease production, the level of active extracellular proteases in the pellicle biofilm supernatant was quantified biochemically (Fig. 5). Consistent with the increase in *bpr* transcription that was observed in more mature biofilms, the level of extracellular proteases in the pellicle supernatant fraction increased during biofilm formation as measured using caesin digestion assays (see Methods; compare Fig. 3 with Fig. 5). At 96 h, exoprotease activity in the extracellular environment was four fold higher than that quantified for the 24 h biofilm (Fig. 5). These biochemical analyses link increases in transcription from the P*bpr*–*gfp* reporter with increased exoprotease activity levels in the biofilm community.

### Exoprotease-producing cells are located at the surface of the mature biofilm

**Fig. 6.**
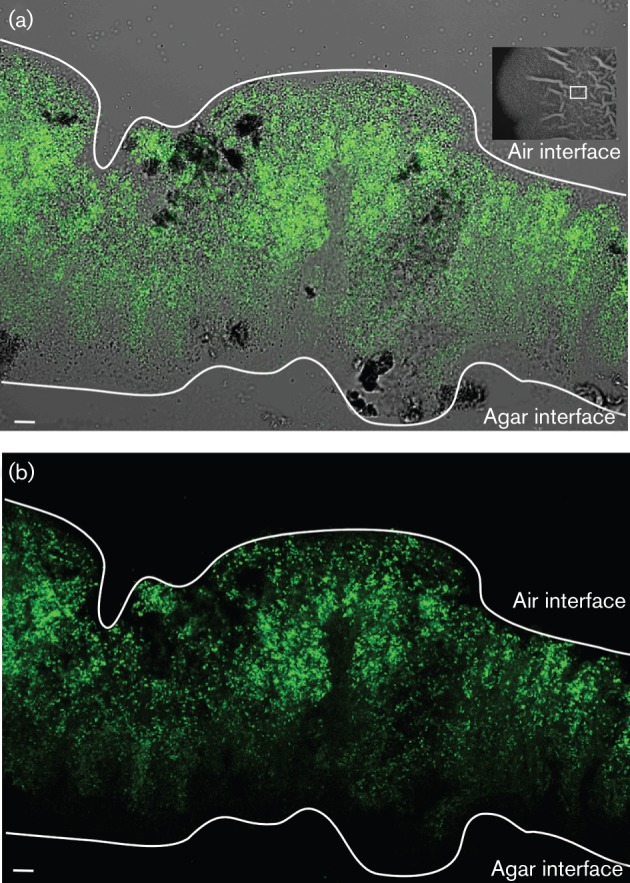
Spatial analysis of *bpr* transcription within the mature biofilm. (a) Bright-field and FITC merged image and (b) FITC image of a 9 µm vertical cross-section of a 48 h colony biofilm harbouring the P*bpr*–*gfp* reporter fusion (NRS3921) detected by confocal microscopy. The top (air interface) and bottom (agar interface) of the colony biofilm are indicated for reference purposes and shown as white lines. In (a), a 48 h colony biofilm is shown and the white box represents the approximate region of the biofilm that was imaged in cross-section. Bars, 10 µm. The images shown are representative of at least three independent experiments during which multiple fields of view were examined.

Flow cytometry and single-cell microscopy analysis of disrupted biofilms allow quantification of the population that express the P*bpr*–*gfp* reporter (Figs 1–4). However, these techniques do not allow analysis of the spatial localization of transcription in the biofilm (Vlamakis *et al.*, 2008). To determine the spatial localization of the exoprotease-producing cells in the biofilm, 48 h colony biofilms formed by strain NRS3921 were cryosectioned as described previously [see Methods and Vlamakis *et al.* (2008)]. Following fixation, the thin layer cross-sections of the biofilm were imaged by confocal microscopy and expression from the P*bpr*–*gfp* reporter was detected. These microscopy analyses confirmed the flow cytometry analysis presented in Figs 3 and 4, which demonstrated that a subpopulation of the cells was highly fluorescent (indicative of high levels of P*bpr*–*gfp* transcription). Retention of the biofilm structure allowed us to determine that this group of cells was found towards the top of the colony biofilm near the air–biofilm interface (Fig. 6). The subpopulation of cells with low levels of GFP was found towards the centre of the biofilm section. It was highly apparent that transcription of the reporter fusion was heterogeneous within the biofilm and that there was structure in the transcription profile with respect to the organization of the mature biofilm.

### The protease-producing cell population overlaps with the matrix-producing cell population

**Fig. 7.**
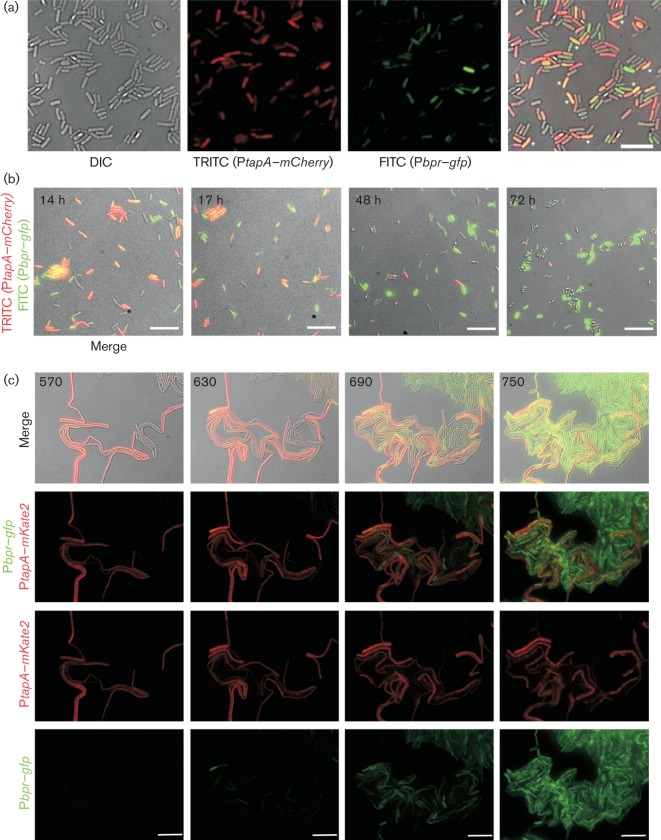
Co-expression of the *bpr* and *tapA* genes. (a, b) Static microscopy of NRS3378 cells harbouring the P*tapA*–*mCherry* and P*bpr*–*gfp* transcriptional reporter fusions extracted from a pellicle biofilm after 24 h growth at 37 °C (a), where the asterisks indicate selected cells for which fluorescence was detected in both the TRITC (false coloured red) and the FITC (false coloured green) channels, for colony biofilms grown at 37 °C for the time (hours) indicated in the upper left-hand corner (b). Bars, 5 µm; the images are representative of multiple fields of view. (c) Microscopy analysis of NRS3921 harbouring P*tapA*–*mKate2* and P*bpr*–*gfp* transcriptional reporter constructs in real-time during microcolony development at 30 °C. Strain NRS3921 was imaged every 15 min. Images from the DIC, FITC (false coloured green) and TRITC (false coloured red) channels are shown above. The time (minutes) is indicated in the upper left-hand corner. Bars, 10 µm.

The analysis reported above allows us to add exoprotease-producing cells to the list of specialized cell types that are found in the developing *B. subtilis* biofilm (Branda *et al.*, 2001; Vlamakis *et al.*, 2008). Previously characterized cell types include cells that are motile, cells that transcribe the *eps* and *tapA* operons needed for biofilm matrix assembly (hereafter matrix-producing cells), and cells that are sporulating (Vlamakis *et al.*, 2008). It has previously been established that motile cells transition into matrix cells and that the matrix-producing cells progress to form endospores at late biofilm stages (Vlamakis *et al.*, 2008). In addition, it has been proposed that matrix production and protease production are mutually exclusive events and that both cell types arise directly from motile cells in response to different environmental signals (Lopez *et al.*, 2009). This has not been tested experimentally and is somewhat at odds with the knowledge that matrix production decreases during biofilm formation (Vlamakis *et al.*, 2008) while exoprotease production increases (Figs 3 and 4) at a time during biofilm formation when motile cells are absent from the biofilm (Vlamakis *et al.*, 2008). Therefore, to define the origin of the exoprotease-producing cells and investigate the relationship between exoprotease production and matrix production, we constructed a dual reporter strain which carried the P*bpr*–*gfp* fusion at the native locus and a P*tapA*–*mCherry* transcriptional fusion at the heterologous *lacA* locus (NRS3378). We examined the prevalence of cells that co-expressed both fusions as indicated by fluorescence in both the FITC (P*bpr*–*gfp*) and TRITC (P*tapA–mCherry*) channels in cells extracted from 24 h pellicle biofilms. As indicated in Fig. 7(a) (asterisks), co-expression from the *tapA* and *bpr* promoter regions was clearly observed within the cells that are false coloured yellow. We next examined expression from each promoter by fluorescence microscopy over a time-course of biofilm formation using cell samples that were extracted from complex colonies (Fig. 7b). The parental strain NCIB3610 was used as a control for microscopy (data not shown). Our analysis demonstrated, as expected, that the proportion of matrix-producing cells was high at early time points of biofilm formation and was lower in the later stages of biofilm development (Fig. 7b, compare 14 h with 72 h) (Vlamakis *et al.*, 2008). Moreover, as described above, the proportion of cells in the biofilm that had transcribed the P*bpr–gfp* reporter fusion increased over time (Fig. 7b). Thus, each transcriptional fusion behaved as expected in the dual reporter fusion strain NRS3378 and the findings suggest that matrix production and exoprotease production are not necessarily mutually exclusive cell states. However, note that the co-expressing cells could represent a transition between one cell state and another or possibly apparent co-expression that is a reflection of the stability of the fluorescent reporter fusions.

### Matrix-producing cells can transition into exoprotease-producing cells

To trace the origin of exoprotease-producing cells and to investigate the relationship between exoprotease production and matrix production in greater detail, we performed real-time fluorescence single-cell microscopy analysis in developing microcolonies (de Jong *et al.*, 2011; Young *et al.*, 2012). Our initial analysis highlighted that the mCherry fluorescent protein was not a suitable reporter protein for the live cell microscopy analysis. Live cell microscopy demands multiple images to be taken and the fluorescence from mCherry was found to be susceptible to rapid photo-bleaching (data not shown). Therefore, strain NRS3921 was constructed where the mCherry reporter was replaced with mKate2, yielding a strain that carried the P*tapA*–*mKate2* and P*bpr*–*gfp* reporter fusions (Table 1). The strain was grown in microscope chambers for up to 13 h, with images acquired every 15 min (see Methods). As expected, P*tapA*–*mKate2* matrix gene expression was bimodal in the developing microcolony and P*bpr*–*gfp* exoprotease gene expression was heterogeneous in the population (Fig. 7c). Moreover, consistent with microscopy and flow cytometry analysis from the time-course of biofilm formation (Figs 3 and 4), transcription from the P*bpr*–*gfp* reporter fusion was observed more frequently at later time points in microcolony development (compare 570 with 750 min time points in Fig. 7c).

**Fig. 8.**
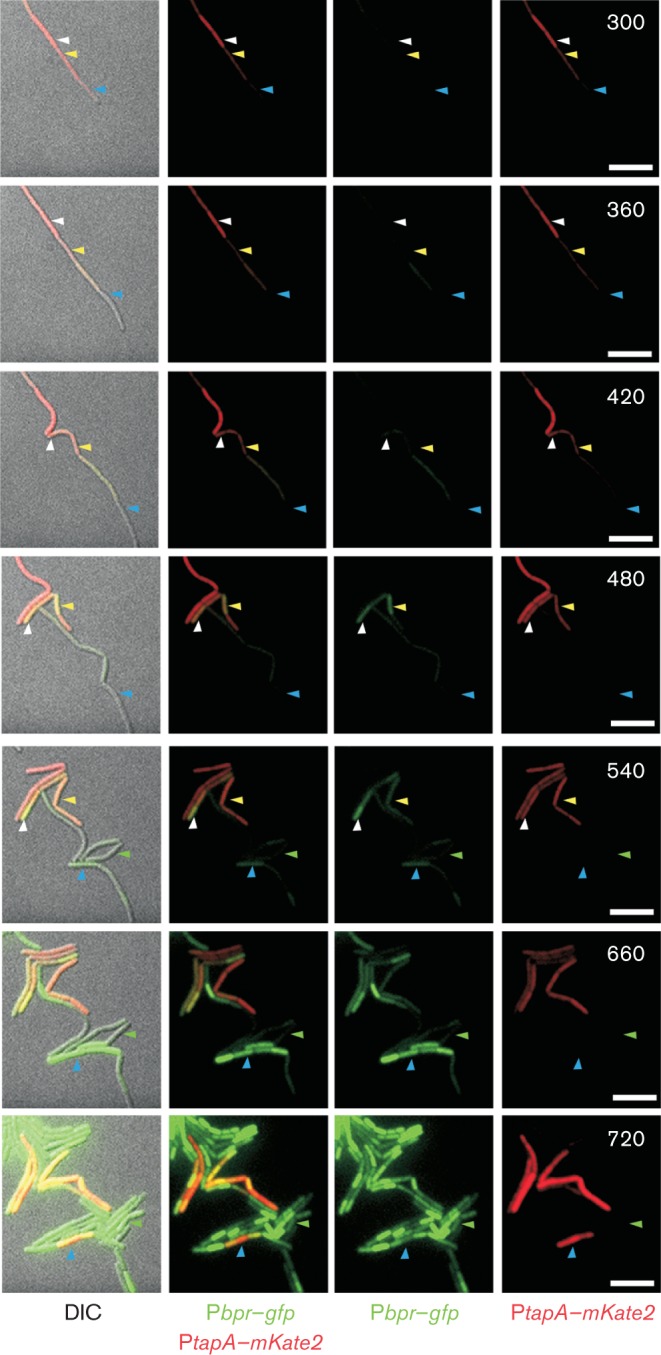
Origin of exoprotease-producing cells. Microscopy analysis of NRS3921 harbouring P*tapA*–*mKate2* and P*bpr*–*gfp* transcriptional reporter constructs during microcolony development at 30 °C. Strain NRS3921 was imaged every 15 min. Images from the DIC, FITC (false coloured green) and TRITC (false coloured red) channels are shown. The white and yellow arrowheads indicate cells that have transitioned directly from matrix production to exoprotease production. The green arrowheads indicate cells that have transitioned directly from a non-fluorescent state to exoprotease production. The blue arrowheads indicate cells that have transitioned from matrix-producing cells to exoprotease production and back to matrix production. The time (minutes) is indicated in the upper right-hand corners. Bars, 5 µm.

The data collected from the live cell imaging were used to trace the origin of matrix-positive cells over several cell cycles. To achieve this we followed multiple cells during division, noting the phenotype as indicated by expression from the reporter fusions. We established that the majority of exoprotease-producing cells arose from cells that had persisted in a non-matrix-expressing state for more than one generation (Figs 7c and 8, cell highlighted by the green arrowheads, and data not shown). However, we established that exoprotease-producing cells were not precluded from arising directly from matrix-producing cells as the transition of a matrix-producing cell into an exoprotease producer was frequently detected (Fig. 8). This is exemplified in Fig. 8 where the white and yellow arrowheads on the micrographs highlight two cells that transition directly from matrix production to exoprotease production over time. In addition, it was observed that exoprotease-producing cells were (infrequently) capable of transitioning back to matrix-transcribing cells. This is demonstrated in Fig. 8 by the blue arrowheads. These findings demonstrate that exoprotease production and *tapA* matrix gene expression are not incompatible events and can exist for a sustained period of time within one cell.

### Concluding Remarks

Here we have studied the prevalence and origin of exoprotease-producing cells in the developing *B. subtilis* biofilm. We have determined that the production of extracellular proteases is correlated with later stages of biofilm formation. This is perhaps due to a role in biofilm dispersal or nutrient acquisition and is consistent with the *in situ* localization of the exoprotease-producing cells at the biofilm–air interface. Note that this is the same region of the mature biofilm where developing spores are located and are therefore perhaps dispersed into the environment (Branda *et al.*, 2001; Vlamakis *et al.*, 2008). Through the use of live single-cell fluorescence microscopy we have defined the origin of exoprotease-producing cells and established that there is not a strict dependence on the phenotype of the parental cell. We noted the development of the exoprotease-producing state from both a matrix OFF state and matrix ON state. Indeed, a matrix- and exoprotease-producing cell state can exist for an extended period of time, demonstrating that they are not mutually exclusive cell states (Fig. 8). The biological significance of having a group of cells that can contribute to the production of the biofilm extracellular matrix and extracellular proteases remains to be elucidated. However, co-production of these molecules may indicate a need to increase nutrient acquisition from the extracellular environment when a sessile lifestyle is adopted. The single-cell analyses techniques used in this study clearly demonstrate the diversity of cell differentiation processes in the biofilm and indicate that, unlike matrix-producing cells that arise only from motile cells (Vlamakis *et al.*, 2008), the origin of exoprotease-producing cells in the population is more flexible. In addition, as matrix-producing cells can transition into exoprotease-producing cells, it will be of interest in the future to determine if exoprotease-positive cells subsequently transition into spore formers. If correct, this would add an additional step to the cell fate lineage previously observed during biofilm development (Vlamakis *et al.*, 2008).
